# Orthorexia nervosa and Instagram: exploring the Russian-speaking conversation around #opтopeкcия

**DOI:** 10.1007/s40519-021-01230-4

**Published:** 2021-06-14

**Authors:** Yana Zemlyanskaya, Martina Valente, Elena V. Syurina

**Affiliations:** 1 Amsterdam University College, Science Park 113, 1098 XG Amsterdam, Netherlands; 2 Faculty of Science, Athena Institute, Vrije Universiteit Amsterdam, De Boelelaan 1105, 1081 HV Amsterdam, Netherlands

**Keywords:** Orthorexia nervosa, Eating disorder, Instagram, Russian-speaking, Social media, Cross-cultural

## Abstract

This mixed-methods study explored the conversation around orthorexia nervosa (ON) on Instagram from a Russian-speaking perspective. Two quantitative data sources were implemented; a comparative content analysis of posts tagged with #opтopeкcия (*n* = 234) and #orthorexia (*n* = 243), and an online questionnaire completed by Russian-speakers (*n* = 96) sharing ON-related content on Instagram. Additionally, five questionnaire participants were interviewed, four of which identified with having (had) ON. Russian-speakers who share ON-related content on Instagram are primarily female, around their late-twenties, and prefer Instagram over other platforms. They describe people with ON as obsessed with *correct* eating, rather than *healthy* or *clean* eating. Instagram appears to have a dual effect; it has the potential to both trigger the onset of ON and encourage recovery. Positive content encourages a healthy relationship with food, promotes intuitive eating, and spread recovery advice. Harmful content, in turn, emphasizes specific diet and beauty ideals. Russian-speaking users mainly post pictures of food, followed by largely informative text that explains what ON is, and what recovery may look like. Their reasons for posting ON-related content are to share personal experiences, support others in recovery, and raise awareness about ON. Two main target audiences were people unaware of ON and people seeking recovery support. The relationship between ON and social media is not strictly limited to the global north. Thus, it may be valuable to further investigate non-English-speaking populations currently underrepresented in ON research.

*Level of evidence:* Level V, descriptive study.

## Introduction

Food consumption plays an undeniable role in the maintenance of physical health, with the potential to increase life expectancy and decrease the risk of chronic disease [1]. Adherence to a healthy diet is, therefore, increasingly more recognized and encouraged in our present society [2, 3]. However, what appears as a virtuous pursuit of improving health may sometimes actually be an unhealthy obsession with nutrition [4]. Literally, orthorexia nervosa (ON) is translated as “correct appetite” to describe a pathological fixation on eating healthy [5]. ON is characterized by an obsession with *quality* of food rather than *quantity*, unlike other eating disorders such as anorexia nervosa and binge-eating disorder [6].

Compared to other eating disorders in the Diagnostic and Statistical Manual of Mental Disorders, fifth edition (DSM-5) [7], ON is perceived as less distressing and more accepted, thus its pathological potential often underestimated [3, 8]. The danger of ON lies in the illusion of total health that encourages the insidious behaviours eventually associated with reduced quality of life [4]. The dietary extremism may neglect essential nutrients and sufficient calories, causing physical complications akin to malnutrition [9, 10]. Positive correlations have been found between ON-type eating behaviours and a number of psychological issues, including negative affect and depressive symptoms, chronic stress, and obsessive–compulsive symptoms [11].

One of the largest unrestricted and free sources of dietary information is social media, where the ‘clean eating’ revolution is exaggerated by its habitual use [3, 12, 13]. Instagram, with over 100 million posts shared as of 2020 [14], has been particularly associated with ON symptoms [15]. It seems that placing significant value on physical appearance and diet are associated with disordered eating attitudes [16, 17]. The selective exposure of diet-endorsing content makes certain eating patterns appear more societally prevalent than they actually are, creating a sense of social pressure and reinforcing problematic behaviours [15, 16]. Furthermore, Instagram provides a limitless source of eminence-based dietary practices [3, 13]. “Fitspiration” accounts promoting health and fitness unknowingly spread problematic messages by condemning excess weight and endorsing disordered eating practices camouflaged as ‘healthy’ [18, 19]. The idea that a stricter diet is ‘better’ is fuelled by the normalisation of food restriction and association of guilt with eating certain foods [18]. Dieting becomes a matter of moral significance where “healthy” eating is socially praised, and “unhealthy” eating feels like a crime [12].

Findings on the relationship between Instagram use and ON are mixed [12]. While some suggest that higher Instagram use is linked to more ON symptoms [15], others propose Instagram may be a supportive platform with positive online communities that encourage recovery [20]. Based on an online survey given to social media users (*N* = 680) that follow health food accounts, an increased predisposition towards ON was associated with greater Instagram use, while other social media platforms lacked evidence of the same effect. Such findings place an emphasis on the influence social media can have on mental health [15].

At present, ON is a growing topic of interest predominantly in the global north with the vast majority of research focused on English-speaking populations [15, 21–24]. This represents a research gap in our understanding of the intercultural differences in the way ON manifests, is experienced, and understood. This is important for a number of reasons. First, gaining insight into socio-cultural differences regarding ON may help in understanding its aetiology. This is particularly relevant for ON, since health and nutrition are greatly dependant on culture. Next, understanding the differences in how ON manifests across cultures may help avoid ethnocentricity in our conceptualization and assessment of ON [11]. One of the countries that has not yet been researched is Russia. The popularity of dieting for health purposes and discourse surrounding ON is growing in Russian social media contexts. For instance, the trend of ЗOЖ (здopoвый oбpaз жизни—*healthy lifestyle*) or ПП (пpaвильнoe питaниe—*correct eating*) are growing massively, predominantly among young adults and especially on social media like Instagram [25]. Moreover, the preoccupation with food-related allergies is particularly growing in popularity, especially through the media where food products are separated into categories of healthy (e.g. organic, free of pesticides, artificial flavouring etc.) versus harmful [26]. According to Neliubina et al. [26], the extremely restrictive eating patterns and consumption of strictly *correct* and clean foods, as well as the relentless search for allergy causes mask what appears to be ON. Research on Russian-speaking users on Instagram could elucidate such underrepresented perspectives, contribute to the understanding of ON, and expand our knowledge of socio-cultural influences on disordered eating.

The aim of this study is to explore the conversation around ON on Instagram from the perspective of Russian-speaking users by investigating *who* posts under the Russian #opтopeкcия, *what* is being posted and *why*.

## Methods

The present study used a mixed-methods design. This consisted of (1) online questionnaires completed by individuals who share content on Instagram under #opтopeкcия, (2) semi-structured interviews with individuals who post about ON on Instagram using #opтopeкcия, and (3) a comparative content analysis of #opтopeкcия vs. #orthorexia posts. See Table 1 for a summary of the methods used for each main research question.

**Table 1:** Main research questions and methods summary

Research question	Methods used
Who interacts with ON on Instagram?	Online questionnaire, interviews
What do Russian-speaking Instagram users post under #opтopeкcия?	Comparative content analysis, interview
Why do Russian-speaking users post about ON on Instagram?	Online questionnaire, interviews

### Online questionnaire

#### Data collection

The questionnaire was created using Qualtrics Survey Software and consisted of four parts: (a) demographic information, (b) social media use, (c) general knowledge and opinions of ON, and (d) ON-related Instagram activity. Russian-speaking users who shared content under #opтopeкcия were sent a link to the online questionnaire via direct message on Instagram. Participants provided written consent before starting the questionnaire.

#### Data analysis

Questionnaire results were analysed using IBM SPSS Statistics version 24. The results were analysed using descriptive statistics, Pearson’s *χ*^2^ test, Fisher’s exact test, and Mann–Whitney *U* test for associations between self-identification with ON and their ON-related knowledge, opinions, and Instagram use. Tests were two-tailed and significance level was alpha 0.05. Open questions were analysed in Excel using open coding.

### Interviews

#### Data collection

Upon completion of the questionnaire, participants were asked to provide their email if they were interested in being contacted for further research. A total of 31 participants were sent an interview invitation, five of which responded and agreed to partake in the interviews. The inclusion criteria were: obtained informed consent, completed questionnaire, and participant 16 years or older. The semi-structured interviews were held in Russian using Whatsapp audio calls and video calls on Skype. Participants gave written consent, permission to record, and verbal consent prior to the interviews. The following topics were discussed: general information about the participant’s Instagram content, motivation and procedure for posting, target audience, and opinion on the influence of Instagram on onset of and recovery from ON.

#### Data analysis

The interviews were transcribed verbatim in Russian and manually coded in English. Relevant quotes were translated into English for publication purposes. The main steps of a thematic analysis were followed [27], but both inductive and deductive measures were implemented throughout. After a familiarisation with the transcripts, a preliminary set of codes was developed and compared to questionnaire findings and previous research to establish consistency. These codes were used for transcript analysis, with the use of inductive coding to allow for the inclusion of emerging codes. Finally, a thematic map of interconnected codes under broader themes was created to identify relationships and reveal patterns.

### Comparative content analysis

#### Data collection

All posts (including captions) shared on Instagram in February 2020 under #opтopeкcия (*N* = 245) and #orthorexia (*N* = 1797) were downloaded. To homogenize the sample sizes, a random subsample of 245 #orthorexia posts was selected, resulting in a total of 490 posts for analysis. Posts shared in languages other than English or Russian (11 posts for #orthorexia and 2 for #opтopeкcия) were excluded from coding. A total of 477 posts were coded, 234 for #orthorexia and 243 for #opтopeкcия.

#### Data analysis

After an overview of the downloaded posts and consultation of literature, a preliminary codebook describing images and the captions was developed and updated during the coding process to include new, unanticipated code categories. The final codebook consisted of 45 codes, which were grouped into five broader themes. Each post was given one code for the most eminent subject of the post. Each caption was also given a code based on its main subject. Findings of the content analysis were reported using descriptive statistics.

### Ethical considerations

The study was tested via the online self-check of the Ethical Committee of the Faculty of Science of VU, Amsterdam. No additional ethical evaluation was required. The participants were informed about their voluntary participation and ability to withdraw during questionnaires and interviews, and informed consent was obtained before participation. All collected data were anonymised and remained confidential and stored on a password-protected Surf-drive storage.

## Results

Results from the content analysis, questionnaire, and interviews are combined into three sections in response to the following questions: (a) Who interacts with ON on Instagram and how? (b) What do Russian-speaking Instagram users post under #opтopeкcия? (c) Why do Russian-speaking users post about ON on Instagram?

### Who interacts with ON on Instagram?

#### Questionnaire sample

A total of 106 responses were recorded, of which 10 were excluded from analysis because of failure to complete the questionnaire. This left 96 respondents, of whom 90 identified with sharing ON-related content on Instagram (“posters”). Identified posters are primarily female, with a mean age of 29 years, mostly born and residing in Russia. They are active, relatively heavy social media users who prefer Instagram over other platforms. About one in three found out about ON via social media. For further demographic information, see Table 2.

**Table 2:** Demographic information of all participants (*N* = 96) and participants who declared to be sharing ON-related content (*N* = 90)

Variables	All participants *N* = 96	Participants who share ON-related content *N* = 90 (93.8%)
Gender, *n* (%)		
Female	89 (92.7)	85 (94.4)
Male	7 (7.3)	5 (5.6)
Age (years), mean (SD)	29 (7.0)	29 (7.0)
Country of birth, *n* (%)		
Belarus	6 (6.4)	6 (6.7)
Kazakhstan	3 (3.2)	3 (3.3)
Latvia	2 (2.1)	2 (2.2)
Moldova	1 (1.1)	1 (1.1)
Russia	68 (72.3)	63 (70.0)
Ukraine	14 (14.9)	14 (15.6)
No response	2 (2.1)	1 (1.1)
Country of residence, *n* (%)		
Azerbaijan	1 (1.0)	1 (1.1)
Belarus	4 (4.2)	3 (3.3)
Czechia	1 (1.0)	1 (1.1)
Denmark	1 (1.0)	1 (1.1)
France	1 (1.0)	1 (1.1)
Kazakhstan	2 (2.1)	2 (2.2)
Latvia	1 (1.0)	1 (1.1)
Lebanon	1 (1.0)	1 (1.1)
Moldova	1 (1.0)	1 (1.1)
Norway	1 (1.0)	1 (1.1)
Poland	1 (1.0)	1 (1.1)
Russia	62 (64.6)	59 (65.6)
Spain	1 (1.0)	1 (1.1)
Turkey	2 (2.1)	2 (2.2)
Ukraine	10 (10.4)	9 (10.0)
United Kingdom	1 (1.0)	1 (1.1)
United States	3 (3.1)	3 (3.3)
No response	2 (2.1)	1 (1.1)
Highest education, *n* (%)		
Secondary education	4 (4.2)	4 (4.4)
Vocational education	6 (6.3)	5 (5.6)
Bachelors	35 (36.5)	33 (36.7)
Masters	31 (32.3)	29 (32.2)
Other	18 (18.8)	18 (20.0)
No response	2 (2.1)	1 (1.1)
Professional status^a^, *n* (%)		
Full-time	40 (41.7)	39 (43.3)
Part-time	36 (37.5)	34 (37.8)
Unemployed	11 (11.5)	9 (10.0)
Unpaid/voluntary	2 (2.1)	1 (1.1)
Student	19 (19.8)	19 (21.1)

^a^Multiple answers were possible

#### Interview sample

A total of five participants who shared ON-related content and used #opтopeкcия on Instagram were interviewed, four of which identified with having (had) ON (“self-identifiers”). All were female, mean age of 29.4 years (SD = 4.4). Three reside in Russia, one in Turkey, and another in Ukraine. Four were employed, one was unemployed.

### Knowledge and opinions about ON

#### Definition of ON

**Fig. 1:**
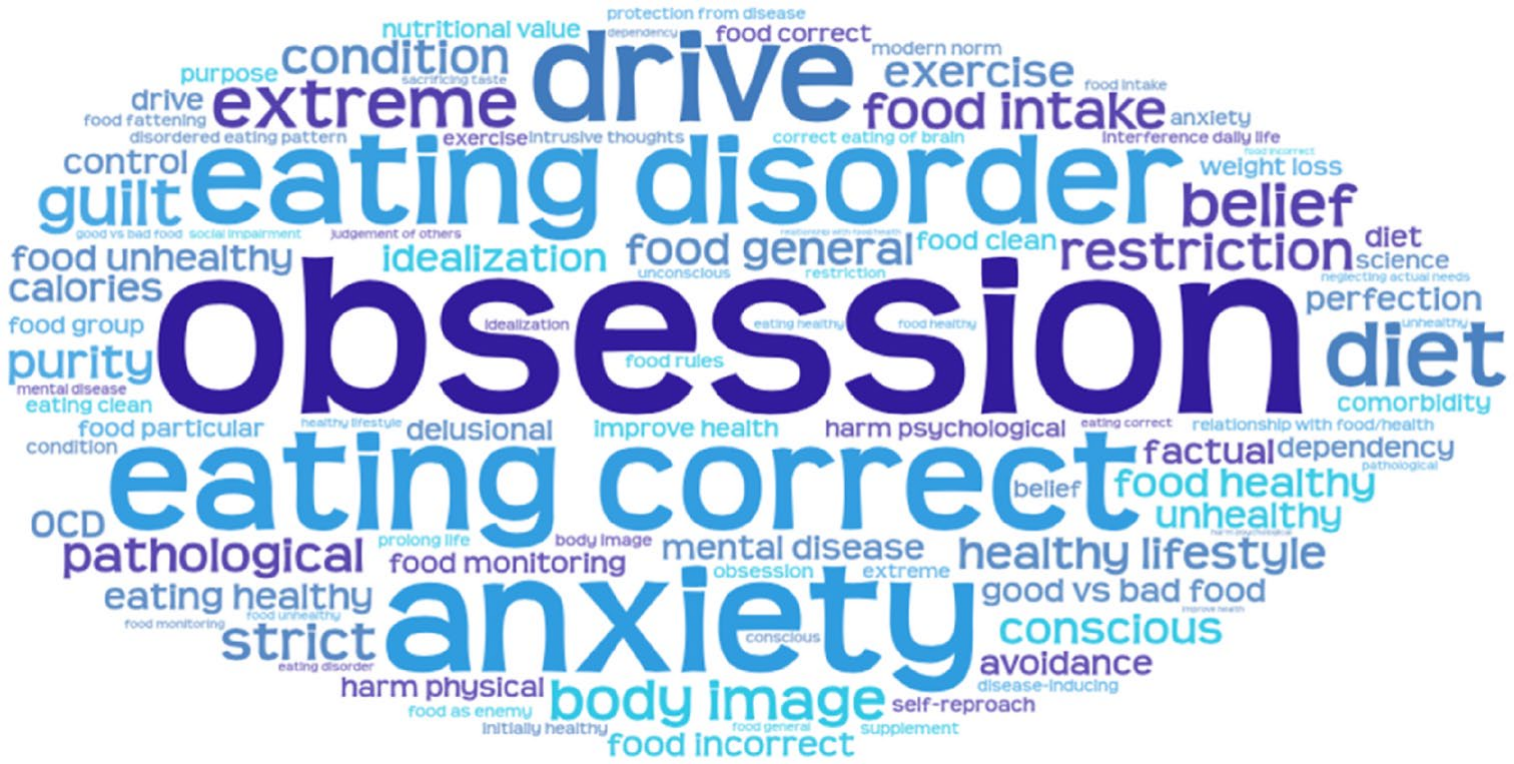
Word cloud of codes for descriptions of ON provided by questionnaire participants

Over half of questionnaire participants described ON as an obsession (54.2%). ON was characterized as an eating disorder by 22.9% of questionnaire participants and all interviewees. Other popular labels were: pathological (13.5%), extreme (13.5%), and a strong drive (16.7%) to eat a certain way. For full overview of the words associated with ON, see Fig. 1.

Eating *correct* was associated with ON by self-identifying interviewees and 32.3% of questionnaire respondents. This was much more frequent compared to eating *healthy* (7.3%). Diet (7.3%), restriction (11.5%), and separating food as good or bad (8.3%) were also mentioned. The biggest negative consequence associated with ON was anxiety (16.7%).

#### ON and Instagram

Most posters found out about ON via social media (32.3%). The vast majority of posters were aware that ON is not an official diagnosis (83.3%) and believed it should be (85.6%). Most posters self-identified with having (had) ON (84.4%) and know someone who may have ON (76.6%).

All interview participants agreed that Instagram has a significant influence on ON but held contrasting views on the extent of Instagram’s support for recovery. Three interviewees believed that content encouraging a positive relationship with food by promoting intuitive eating or giving advice on how to recover can be beneficial. However, at the same time, three of five believed that Instagram did not have enough helpful content and that viewers should seek professional help: “*It is essential that you look for a psychologist, psychotherapist and so on. Because without him*, *my account will not save you… My account is purely like crutches at the beginning, you will need to release them and work seriously*”(P2).

The forceful nature of dietary ideals was believed to negatively impact the mental health of viewers and contribute to the onset of ON: “*On Instagram I became acquainted with correct eating. I ate correctly for about 2 years, then there was more talk about healthy eating… so it led me to orthorexia.* (Instagram) *has a lot of influence. Because there’s this ideal picture and no one knows the truth behind those people who are eating correctly*” (P1). The propaganda of specific diets makes users believe that there is a ‘right’ way to eat, causing viewers to feel fear and avoidance to foods considered unacceptable: “*these bloggers which propagandize correct eating or, even better, their own diets… In turn* (the posts) *intimidate people.*” (P3). Additionally, excessive amounts of time spent on Instagram and the role of popular figures were also suggested as influencing factors in the development of ON: “*people spend a lot of time* (on Instagram), *there are so-called opinion leaders who have many followers and often people brag a little about their correct eating, encouraging everyone to it too.*” (P4).

#### Use of social media

For posters from the questionnaire sample, the most popular and frequently used platform was Instagram. The majority spend 1–3 h on social media per day, with a relatively active presence (mean of 7.1 on a scale of 1 to 10; 1 = being a passive user, 10 = being an active user). No significant difference was revealed between those who self-identified with having (had) ON (“self-identifiers”) and those who did not, for their time spent on social media (Fisher’s exact test; *p* = 0.872) and activeness on social media (Mann–Whitney *U* test; *p* = 0.606).

Table 3 presents Instagram use for all questionnaire participants in total, as well as a comparison of self-identifiers versus non-self-identifiers. Fisher’s exact tests revealed no significant differences for questionnaire participants’ self-identification with ON, in relation to their sharing of ON-related content (*p* = 1.000), searching for ON-related hashtags (*p* = 0.400), following ON-related hashtags (*p* = 1.000), and following ON-related accounts (*p* = 0.215).

**Table 3:** Cross tabulation of Instagram use for self-identifiers from questionnaire sample

Variable	Self-identify with having (had) ON		
	Yes *N* = 80 (83.3%)	No *N* = 15 (15.7%)	Total *N* = 96
Share ON-related content, *n* (%)			
Yes	76 (95.0)	14 (93.3)	90 (93.8)
No	2 (2.5)	0 (0)	2 (2.1)
No response	4		
Use #opтopeкcияs, *n* (%)			
Yes	75 (93.8)	14 (93.3)	89 (92.7)
No	–	–	–
No response	7		
Content shared on Instagram^a,b^, *n* (%)			
Food	14 (17.5)	1 (6.7)	15 (16.7)^a^
People	27 (33.8)	2 (13.3)	29 (32.2)^a^
Text	29 (36.3)	10 (66.7)	39 (43.3)^a^
Other	29 (36.3)	5 (33.3)	34 (37.8)^a^
Searching for ON-related #, *n* (%)			
Yes	35 (43.8)	8 (53.3)	43 (44.8)
No	44 (55.0)	6 (40.0)	50 (52.1)
Follow ON-related #^c^, *n* (%)			
Yes	6 (7.5)	1 (6.7)	7 (7.3)
No	29 (36.3)	7 (46.7)	36 (37.5)
Follow ON Instagram accounts, *n* (%)			
Yes	26 (32.5)	2 (13.3)	28 (29.2)
No	53 (66.3)	12 (80.0)	65 (67.7)

^a^Responses only by participants who share ON-related content (*N* = 90)

^b^Multiple answers possible

^c^Responses only by participants who search for ON-related hashtags (*N* = 43)

### What do Russian-speaking Instagram users post under #opтopeкcия?

Of the total 234 posts tagged with #opтopeкcия, 53.5% represented food, 19.8% people, 13.6% text, and 13.2% was ‘other’ (Table 4). In comparison, the 243 #orthorexia posts consisted of 52.1% text, 25.6% people, 20.1% food, and 2.1% was ‘other’. A *χ*^2^ test was used to compare the two hashtags, revealing significant differences between the proportions of the code categories, *χ*^2^ (3, *N* = 477) = 443.72, *p* < 0.001.

**Table 4:** Comparative content analysis of #orthorexia (*N* = 234) versus #opтopeкcия posts (*N* = 243)

Code	#opтopeкcия *N* (% of category total)	#orthorexia *N* (% of category total)
Food, *n* (%)	130 (53.5)*	47 (20.1)*
Drink	3 (2.3)	1 (2.1)
Processed, savoury food	79 (60.7)	19 (40.4)
Food and drink	2 (1.5)	4 (8.5)
Fruit and/or vegetable	4 (3.1)	5 (10.6)
Pasta	3 (2.3)	1 (2.1)
Salad	5 (3.8)	4 (8.5)
Sweet food	34 (26.2)	13 (27.7)
People, *n* (%)	48 (19.8)*	60 (25.6)*
Body parts	2 (4.2)	2 (3.3)
Exercise	1 (2.1)	2 (3.3)
Face	1 (2.1)	3 (5)
Group	2 (4.2)	4 (6.7)
Individual	39 (81.3)	37 (61.6)
Mirror selfie	3 (6.3)	12 (20)
Other, *n* (%)	32 (13.2)*	5 (2.1)*
Animal	0 (0)	1 (20)
Art	1 (3.1)	1 (20)
Blank	20 (62.5)	0 (0)
Object	3 (9.4)	1 (20)
Room	2 (6.25)	1 (20)
Scenery	6 (18.8)	1 (20)
Text, *n* (%)	33 (13.6)*	122 (52.1)*
Anti-diet	3 (9.1)	11 (9)
Comparison	0 (0)	5 (4.1)
Event advertisement	6 (18.2)	1 (0.8)
Informative	15 (45.5)*	30 (24.6)*
Meme	0 (0)	3 (2.5)
Personal experience	4 (12.1)	5 (4.1)
Personal opinion	1 (3)	1 (0.8)
Question	0 (0)	4 (3.3)
Supportive	4 (12.1)*	62 (50.9)*

For #opтopeкcия and #orthorexia, most of the ‘food’ posts were savoury, processed food (defined as food that has been industrially processed and packaged). Significant differences between the two hashtags were found for the type of ‘text’ posts, *X*^2^ (3, *N* = 111) = 56.11, *p* < 0.001. Most ‘text’ for #opтopeкcия was *informative*, explaining what ON is and what recovery may look like. While for #orthorexia it was mostly *supportive*, providing emotional recovery support. The captions matched the content of the posts (Table 5). Captions for #opтopeкcия were mainly *food logs* (47.7%): tracking consumed food or drinks for personal record or sharing with viewers. While for #orthorexia captions were mostly *supportive* (18.8%): emotional assistance using motivational references.

**Table 5:** Comparative content analysis of #orthorexia (*N* = 234) versus #opтopeкcия captions (*N* = 243)

Caption code	#orthorexia *N* (%)	#opтopeкcия *N* (%)
Advertisement (for other social media channel or blog)	11 (4.7)	8 (3.3)
Anti-diet	26 (11.1)*	7 (2.9)*
Personal challenges	22 (9.4)*	11 (4.5)*
Exercise	5 (2.1)	3 (1.2)
Food log	17 (7.3)*	116 (47.7)*
Informative	21 (9.0)	30 (12.3)
Intuitive eating	5 (2.1)	0 (0)
No message	33 (14.1)	33 (13.6)
Other	4 (1.7)	1 (0.4)
Personal experience	20 (8.5)	18 (7.4)
Physical symptoms	2 (0.9)	0 (0)
Recipe	4 (1.7)	4 (1.6)
On self-acceptance	20 (8.5)*	4 (1.6)*
Supportive	44 (18.8)*	8 (3.3)*

*Significant difference between #opтopeкcия and #orthorexia sample, *p* < 0.01

For interview participants, the main type of post was self-taken photos of oneself, followed by photos of food: “*photos of food, it clearly attracts attention. A person likes the look of a dish and wants to cook it. I choose photos of myself because, let’s say, photos that are faceless do not evoke such a reaction like your personal photos, even if it is not about you*” (P4).

### Why do Russian-speaking users post about ON on Instagram?

Users’ reasons to post were explored mostly in the interviews, and partly in the questionnaire. The questionnaire findings in this section are based on 34 (of total 96) respondents who specified their reasons for posting their chosen ON-related content. In line with the motivations to share ON content, two common target audiences were identified in both interviews and questionnaires: (1) people with no specific interest in ON, typically unaware of the harm of their eating behaviours, and (2) people with a specific interest in ON, usually seeking help for their disordered eating. No significant differences were found using Fisher’s exact tests for questionnaire participants’ self-identification with ON and their target audience (*p* = 0.468).

Most of the interviewees were motivated to post ON-related content by a desire to share their personal experience, including their challenges and insights from living with ON. Their accounts were a diary for sharing day-to-day feelings and recovery-related developments: *“I didn’t choose this* (content) *because I wanted to make my account a guide, but rather my account was like a diary, I would write things like ‘I feel bad today, this is why’… after some time into recovery, I would write ‘I did so and so to feel better, I advise you to do the same’”* (P2). Similarly, 26.5% of the 34 questionnaire participants were inspired to post by their personal experiences and opinions about ON.

Some interviewees expressed their social identity by explicitly stating that they felt a connection to particular communities, either: “*anti-diet dieticians*” (P5), or a specific recovery group: “*I belong to a group where people search for advice on how to get out of this condition when they feel bad*” (P2). In turn, users are united through their shared value of helping others who are struggling in similar ways to how they have struggled before, so they target people who are actively seeking help: “*if people search for* (#opтopeкcия), *it means that they need this information…they probably have an eating disorder and either search for how to get out of it, or search for more evidence of what they are doing correct in their self-restriction*” (P1). The participants felt like their first-hand experiences made their information reliable and valuable: “*people can relate to me as a source of information… Since I went through this, I got out of this…you can listen to me because I have come a long way*” (P3).

All interviewees believed there was a lack of awareness surrounding ON on Instagram and agreed on the importance of recognizing ON as a restrictive eating disorder: “*ON is an eating disorder and few understand this…any nutrition in which we start to in some way restrict ourselves, is in any case an eating disorder*” (P1). Most interviewees also wanted to elucidate the reality of ON and show viewers that it is often inconspicuous: “*I want to tell people that* (ON) *happens, that it is real… many think that all eating disorders including orthorexia* (happen to) *others. And that…it’s ****not**** like a rash on skin;* (it) *is unnoticeable*” (P5). They explained that some people are not aware that their eating behaviours are unhealthy and aimed to reach this particular audience with the hope of helping them understand the potential harm of their eating behaviours: “*I set up the audience on those that restricting but do not consider themselves sick…because I’m one of those that were happy…with my lifestyle and it turned out that I was sick*” (P2). The goal of informing others was predominant among the 34 questionnaire respondents, 67.6% of whom focused on educating their followers on both ON in particular and eating disorders in general, revealing common misconceptions, and providing informative recovery support.

## Discussion

This study used a mixed-methods design to map the ON–Instagram relationship according to Russian-speaking users based on the following three questions: (a) Who interacts with ON on Instagram and how? (b) What do Russian-speaking Instagram users post under #opтopeкcия? (c) Why do Russian-speaking users post about ON on Instagram?

Identified posters are primarily female, with a mean age of 29 years, mostly born and residing in Russia. They are active, relatively heavy social media users who prefer Instagram over other platforms. About one in three found out about ON via social media.

Russian-speaking users tend to define ON as an obsession with *correct* eating, rather than *healthy* or *clean* eating as is encountered in English literature [6, 23]. This central linguistic difference foreshadows a distinct conceptualisation of ON, framing its occurrence as a partial product of social influence rather than a sole desire to improve health. According to Hanganu-Bresch [12], ON lies on a spectrum of two opposing and morally-infused eating practices on each extreme; healthy and unhealthy eating, with the former lining up neatly with social ideals and the latter frowned upon as a wrongdoing. This focus on moral significance appears more obviously in the Russian emphasis on *correct* eating (literally translated from пpaвильнoe питaниe). While English may suggest ON as a perfecting of diet based on nutritional value, Russian emphasizes more the distinction of a moral correct and incorrect, where ‘orthorexic’ behaviours are led by the striving for a more virtuous position in the social hierarchy. These socially desirable restrictions translate into an unshakeable obedience to a self-imposed and *correc*t diet, highlighting the idiosyncrasy and subjectivity of the food rules one specifically deems as appropriate. Regardless of the motivation behind one’s diet or the type of diet itself, however, ON was identified as an *obsession* by over half of questionnaire participants, in line with previous English-speaking literature [20, 23].

Four main categories encapsulate ON-related content on Instagram: food, people, text, and other. Food, generally savoury and processed, was shared the most by Russian-speaking posters, as per captions, which were mostly food logs. Pictures containing text were largely informative. Santarossa et al. [20] reported that the predominant type of content found on Instagram in their analysis of the #orthorexia conversation was food (68% of 145 posts), in line with the present findings for the Russian-speaking sample (53.5% of 243 pictures), but not the English-speaking sample (20.1% of 234 pictures). Since Santarossa et al. [20] downloaded #orthorexia posts in October 2016, compared to March 2020 of the present study, the differences may represent temporal changes in the ON phenomenon. While Russian-speakers still currently share predominantly informative, food-related posts, older and larger English-speaking communities are perhaps evolving into more supportive, text-type content. Another explanation for the differences in content may be the distinct ways motivations to post on Instagram are expressed. The predominant publication of food-related posts by Russian-speaking users may indicate the focus of raising awareness through a more intimate lens of experiential advice gathered through personal experiences, rather than English-speakers’ text-based and abstract support, usually in the form of motivational quotes.

In the present study, the purpose of users’ accounts can be encapsulated into three main motivations: (1) sharing personal experiences of ON, (2) raising awareness about ON both for recognition among the general public and to help dieters understand the potential harm of their restriction, and (3) support users with experiential knowledge and recovery advice. Two types of target audiences were identified: (1) people who are unaware of ON and the potential harm in their own eating behaviours, and (2) people who are seeking help for their own recovery.

Interview participants believed that Instagram has a significant influence on both the development of and recovery from ON. This dual effect is evident in the disagreement between findings of Turner and Lefevre [15] and Santarossa et al. [20], with the former associating Instagram with the onset of unhealthy behaviours, while the latter observing Instagram as support to one’s recovery journey. Positively influencing factors include posts that support others by raising awareness, provide recovery advice, and promote intuitive eating [20]. Users with intentions to help their viewers, such as the interviewees of the present study, encourage a positive relationship with food. Conversely, caution should be taken when considering Instagram as a tool for support, as the interviewees identified diet-endorsing content on Instagram as a causative factor for ON, and the content analysis revealed a predominant appearance of food posts.

Although Turner and Lefevre [15] found a significant relationship between heavier Instagram use and greater risk of ON symptoms, the present study found no significance between participants’ identification with ON and Instagram use. This could be because this study did not measure ON symptoms but rather used the self-reported and closed question of whether the participants self-identified as having ON or not. Moreover, perhaps Instagram is not associated with the development of ON for Russian-speaking populations due to, for example, heavier use of other social media platforms like Vkontakte or YouTube; or the comparatively smaller ON Instagram community.

### Strengths and limits

This study benefits from the implementation of three data collection methods and the simultaneous use of qualitative and quantitative analyses. However, the small interview sample does have limitations; it is biased to people who identify with having (had) ON and may not be generalisable. Other perspectives such as those of health professionals could be valuable too. Furthermore, despite the depth of the content analysis, it only included a month’s worth of posts. Sampling #opтopeкcия posts over a longer period of time may be valuable to monitor potential changes and increase generalisability. Finally, the interconnectivity of users and use of English hashtags by Russian-speakers makes it difficult to completely separate socio-cultural differences. It is, therefore, unclear whether it is “Western” norms that influence the Russian populations, or inherent similarities in the both cultures that lead to similar disordered eating attitudes.

Regarding future research, it may be valuable to dive deeper into the Instagram recovery community to obtain a more detailed understanding of how the interactions are taking place online, and how the content affects the target audiences. Finally, as the #orthorexia creates limitations by narrowing down the population to individuals who are already aware of it, another suggestion is to explore related hashtags around ON, such as #correcteating or #healthyeating, to gain a more complete perspective of ON on social media.

## Conclusion

There are apparent differences and important similarities between Russian- and English-speaking Instagram users in their conceptualisation of ON that present potentially valuable additions to the understanding of socio-cultural influences on disordered eating. On the one hand, Russian-speakers relating ON to *correct* eating emphasizes the role of social-desirability in the perfecting of one’s diet. The Russian-speakers’ predominantly share informative content about ON and recovery, rather than English-speakers’ supportive content, which suggests a more personal approach of helping others through personal experience and advice. On the other hand, the identification of ON as an *obsession*, whether with clean or correct eating, is a part of most current literature. Further, Instagram appears to have a dual effect on ON, calling for further research on its potential to trigger the onset of unhealthy behaviours as well as inspire and encourage recovery.

### What is already known on this subject?

Research on the relationship between orthorexia nervosa (ON) and Instagram shows mixed results: some suggest that higher Instagram use is linked to increase ON symptoms while others propose Instagram as a supportive platform with the potential to help recovery. Currently, ON research is a topic of interest predominantly in the global north with a main focus on English-speaking populations. Therefore, the present study is an exploration of potential differences in how ON manifests, is experienced, and understood in other cultures, particularly in Russia where the popularity of dieting and discourse around ON are growing.

### What this study adds?

The present study supports the identification of ON as an *obsession*, as often mentioned in previous literature. However, while English-speakers defined ON as an obsession with clean or healthy eating, Russian-speakers related ON to *correct* eating, emphasizing the perceived social influence or pressure on one’s diet particularly via social-desirability and the idea that there are “right” and “wrong” ways to eat. Additionally, Russian-speaking Instagram users shared content that was predominantly informative, and focused on sharing personal and experiential advice to help their viewers. As opposed to English-speaking users who shared predominantly supportive content that was impersonal and motivational. Finally, Instagram appeared to hold both the potential to trigger unhealthy eating behaviours as well as act as support system for those in recovery, which calls for further research on the influence of social media on ON.

## Data Availability

The datasets generated during and/or analysed during the current study are available from the corresponding author on reasonable request.
